# An Innovative Technique of Iris Positioning Using Digital Photographs for Custom Ocular Prosthesis: A Case Report

**DOI:** 10.7759/cureus.58717

**Published:** 2024-04-22

**Authors:** Karthikeyan Palanisamy, Praveen Rajagopal, Grazina Fernandes, Meena Aras, Vidya Chitre

**Affiliations:** 1 Department of Prosthodontics and Crown and Bridge, Goa Dental College and Hospital, Panaji, IND

**Keywords:** digital photography, rehabilitation, maxillofacial prosthesis, iris positioning, custom ocular prosthesis, ocular defect

## Abstract

Trauma, cancer, and congenital abnormalities are the three main causes of eye loss. A person's personal and professional life is left with functional, aesthetic, and psychological problems when they lose one eye. A customized ocular prosthesis made of heat-cured polymethylmethacrylate can be used to restore an eye defect. Fabrication of the customized ocular prosthesis has multiple steps including scleral plank fabrication, iris positioning, and characterization. This article's primary goal is to explain a novel method for iris positioning for better cosmetic outcomes.

## Introduction

Humans depend on their eyes for a variety of functions, including vision and emotional expression. Losing an eye can have negative effects on a person's physical, social, and mental health. Eye loss and removal can result from trauma, pathological conditions, or congenital defects [1]. Evisceration, enucleation, and exenteration are the surgical methods used to remove an eye.

Enucleation is also recommended for the treatment of painful blind eyes, intraocular tumors, and serious, potentially fatal eye infections [2,3]. Post-enucleation socket syndrome is the collective term for conditions that occur after eye enucleation, including superior sulcus deepening, ptosis, ectropion, enophthalmos, and laxity of the eyelids [4]. This is caused by a decrease in orbital volume and displacement of intraorbital structures. Ocular prosthesis is the only noninvasive therapy option available for post-enucleation socket syndrome to restore and rehabilitate these ocular abnormalities.

Ocular prostheses come in two varieties: ready-made (stock) and custom-made. A personalized ocular prosthesis can create eye movement that appears more natural and adapts better to the tissue bed for the patient's comfort than a ready-made prosthesis [5]. Additionally, it removes spaces surrounding fitting surfaces that could irritate and perhaps infect mucosa. Accurate impression taking, wax try-in, iris alignment, eye painting, and prosthesis fitting are typically steps in the traditional prosthetic process. Since there are numerous clinical methods for creating ocular prostheses, the purpose of this research was to describe the simple method for the iris alignment.

## Case presentation

**Figure 1:**
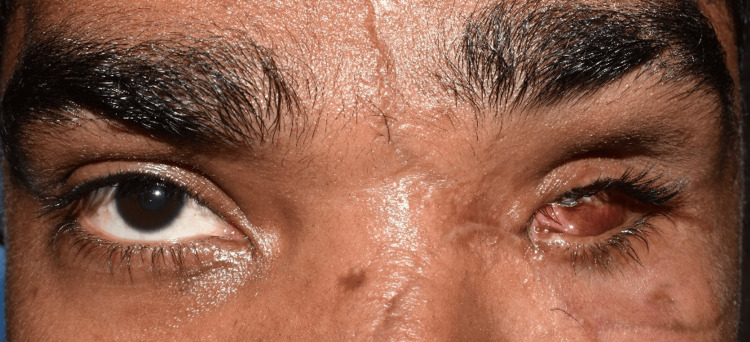
Pretreatment photograph showing an eviscerated left eye socket

A 26-year-old male reported to the Department of Prosthodontics for rehabilitation of his missing eye. While eliciting history, it was found that the patient had undergone evisceration surgery due to a motor vehicle accident. Clinical examination revealed intact eyelids and sufficient supportive tissue (Figure 1). Hence, it was decided to give a custom-made ocular prosthesis.

Custom tray fabrication

**Figure 2:**
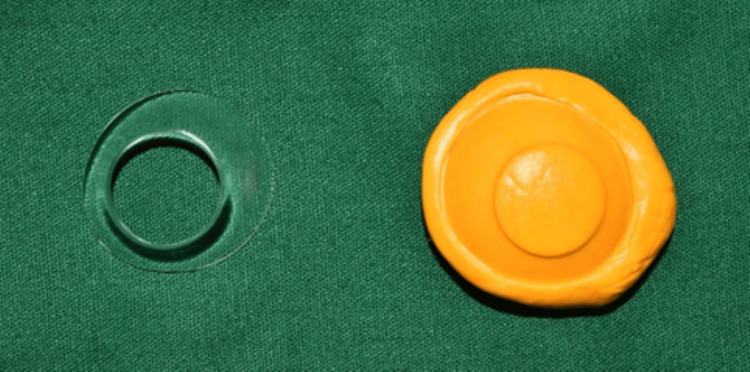
Putty index of the patient’s conformer made using additional silicone

Custom tray for the impression procedure was made with the help of a conformer (Figure 2). Using the outline of the conformer, the putty index was made, and a custom tray was created using autopolymerizing polymethyl methacrylate (PMMA) (DPI, India). After the tray was trimmed and polished, it was tried-in to assess whether there were any overextensions. Any overextensions observed were trimmed, and the tray was repolished.

Impression-making procedure

**Figure 3:**
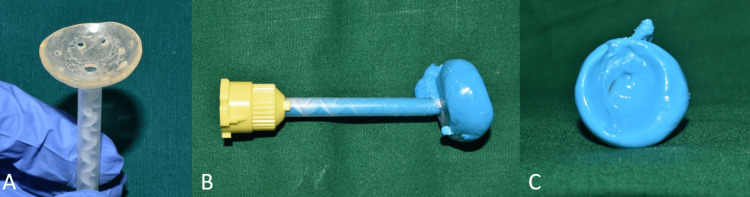
Impression procedure (A) The custom tray was perforated and attached to the automixing tip; (B) an empty socket impression was made with light body, using the automixing tip as a handle; (C) intaglio surface without any voids

The custom tray, perforated for the easy flow of the impression material, was attached to the automixing tip using cyanoacrylate resin (Figure 3A). The socket impression was made using light body impression material (Elite HD+, Zhermack SpA, Badia Polesine, Italy) (Figure 3B). While making the impression, the patient was asked to maintain a straight gaze at a distant object. The impression was inspected for voids and irregularities (Figure 3C).

Fabrication of the scleral wax pattern

**Figure 4:**
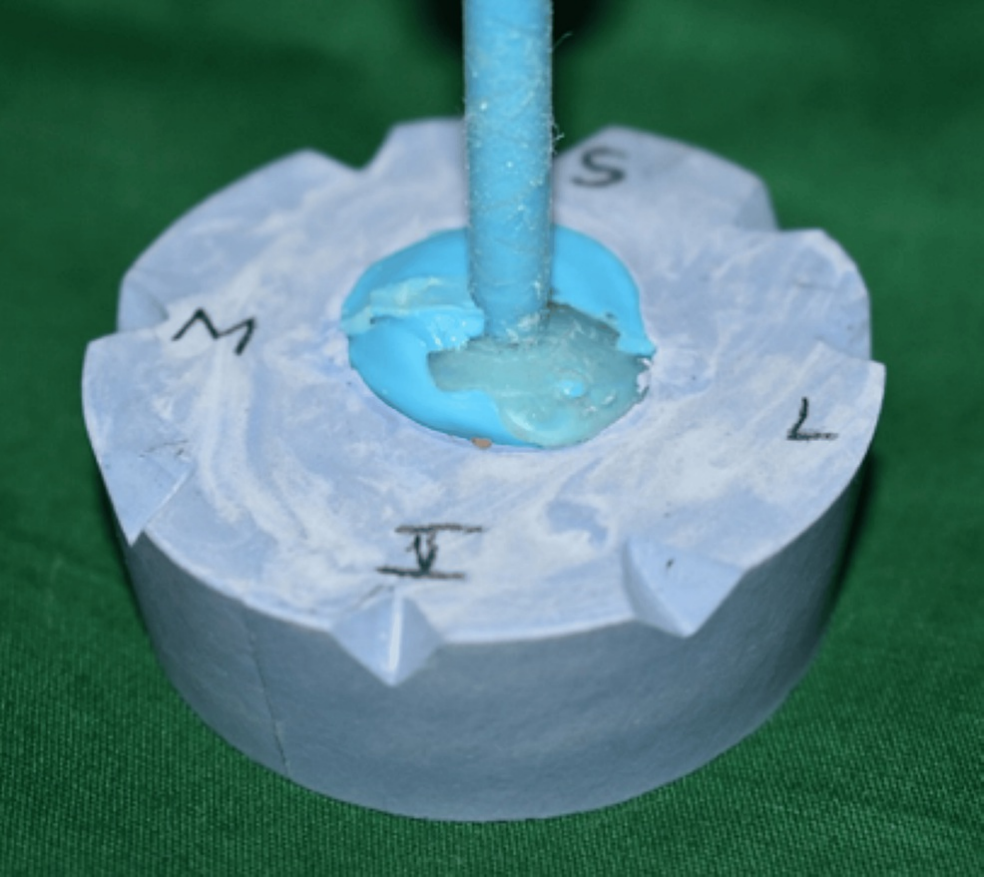
Fabrication of the master cast using the two-pour split cast technique, and the orientation of the cast is marked for the wax pattern orientation in the patient’s eye

**Figure 5:**
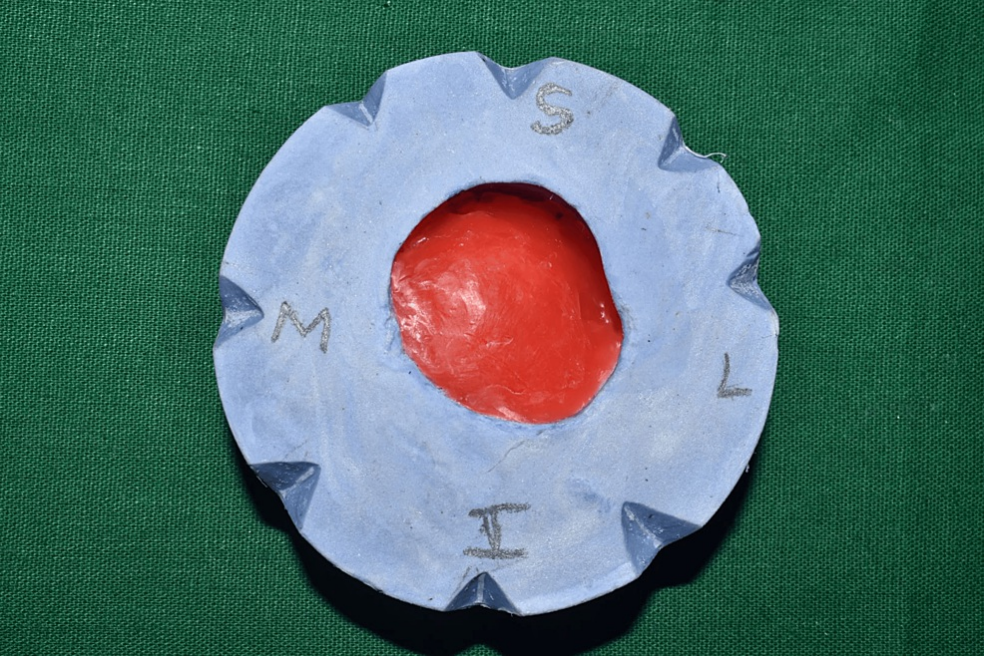
Fabrication of the wax pattern

Using a two-pour cast method, the impression was boxed and poured with type III dental stone (Kalabhai, India) (Figure 4). Melted modeling wax was poured into the master cast after it had been lubricated with a separating medium to make a wax pattern (Figure 5). After being smoothed out, the wax pattern was tried into the patient's enucleated socket. The patient was asked to make all eye motions to assess the wax conformer's retention. Wax pattern adjustments were made until the eyelid closed and opened appropriately.

Iris positioning

**Figure 6:**
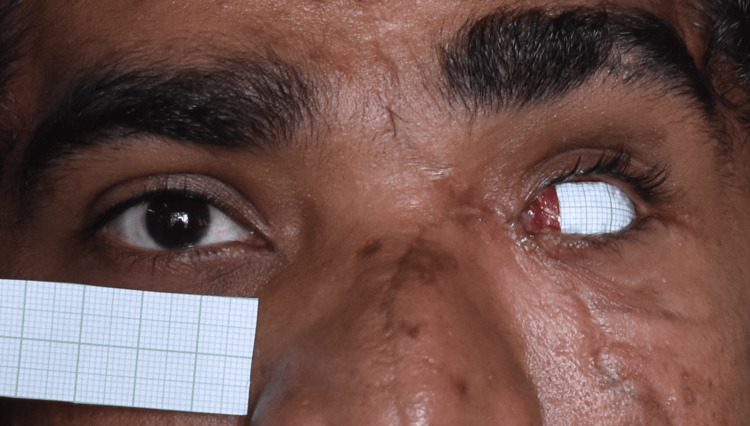
Adhesion of the graph sheet to the wax pattern using wax

**Figure 7:**
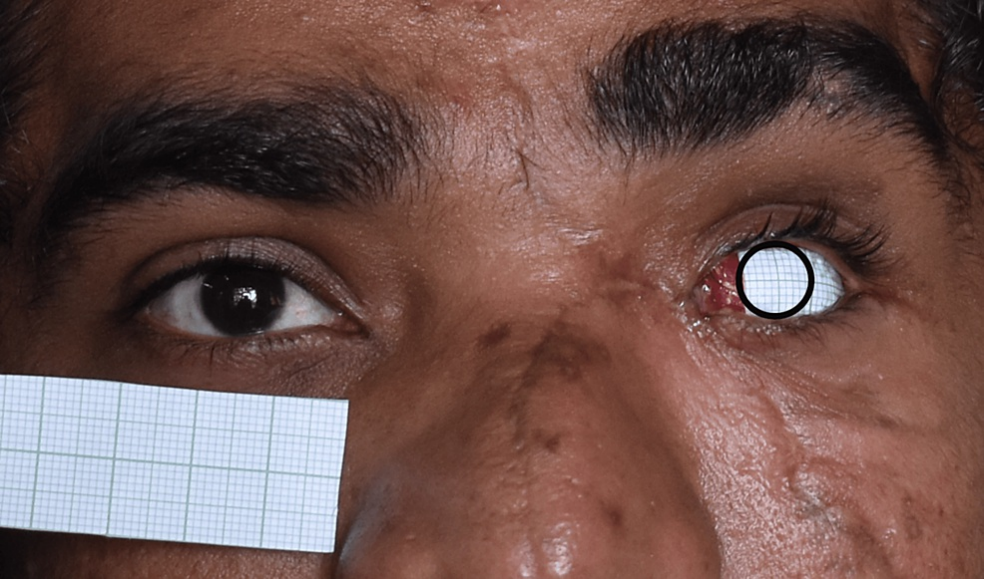
Transfer of the contralateral eye iris measurement using a photo-editing tool

**Figure 8:**
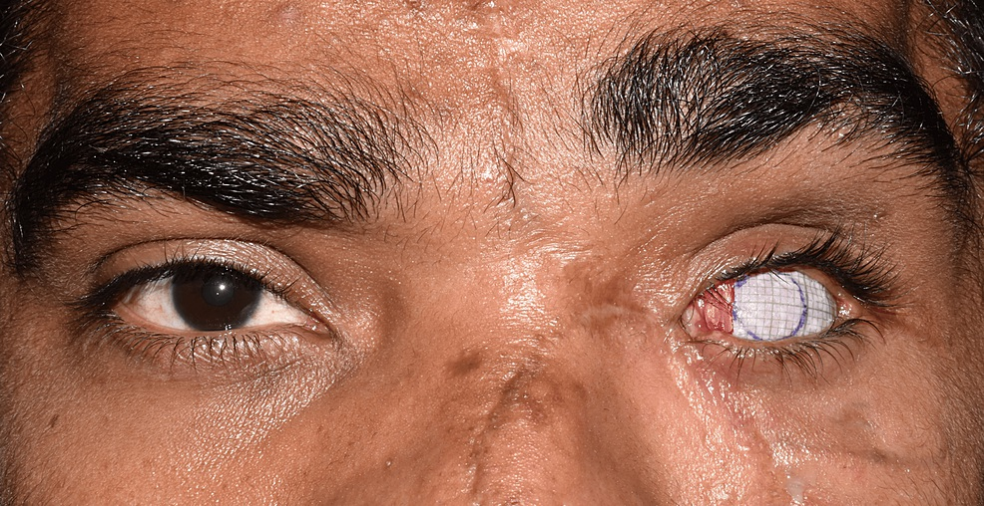
Marking of the contralateral eye iris dimension and position in the graph sheet, and verified

**Figure 9:**
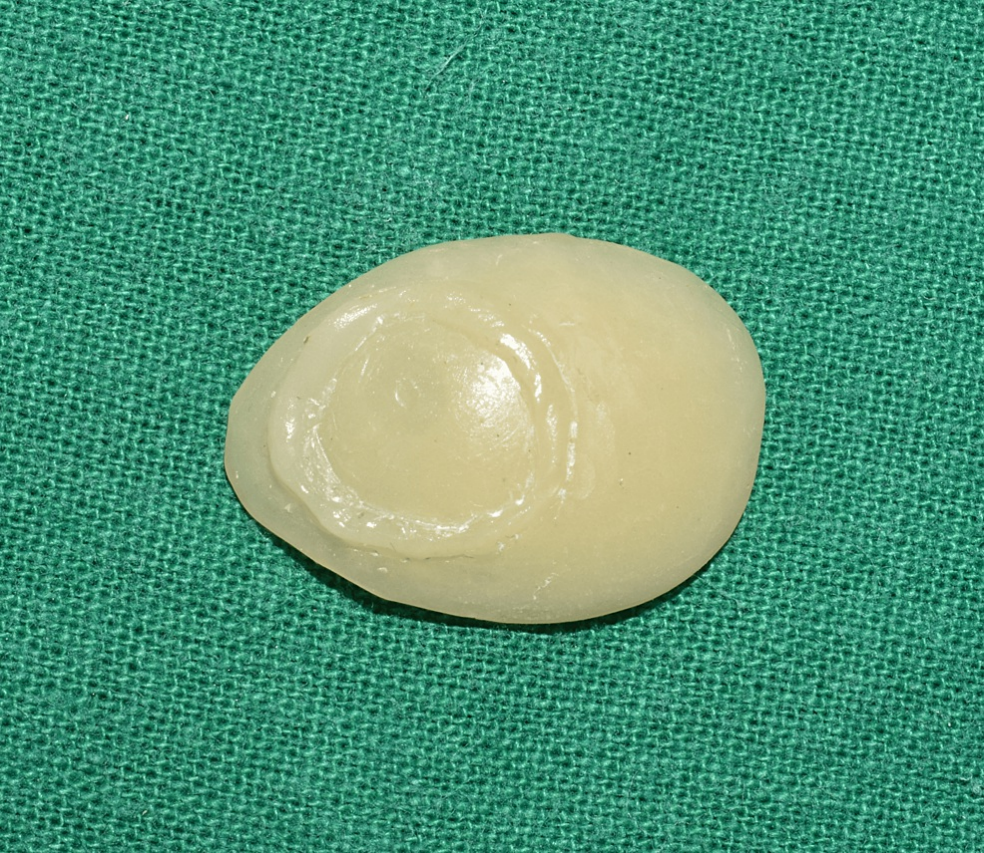
Marking of the iris dimension and position visible in the acrylic blank.

A graph sheet was adhered to the wax pattern (Figure 6), and a digital photograph was taken, and a digital photo editing software tool was employed to meticulously transfer the contralateral eye iris measurements to the graph sheet. Then, this exact measurement was transferred to the graph sheet in the wax pattern (Figure 7). This technique yields accurate measurements, guaranteeing harmonious alignment and a lifelike appearance for the finished prosthesis (Figure 8). Wax is carefully added to the wax pattern using the marks on the graph sheet, making sure that the modifications are visible in the acrylic blank (Figure 9).

Acrylization of the scleral blank

**Figure 10:**
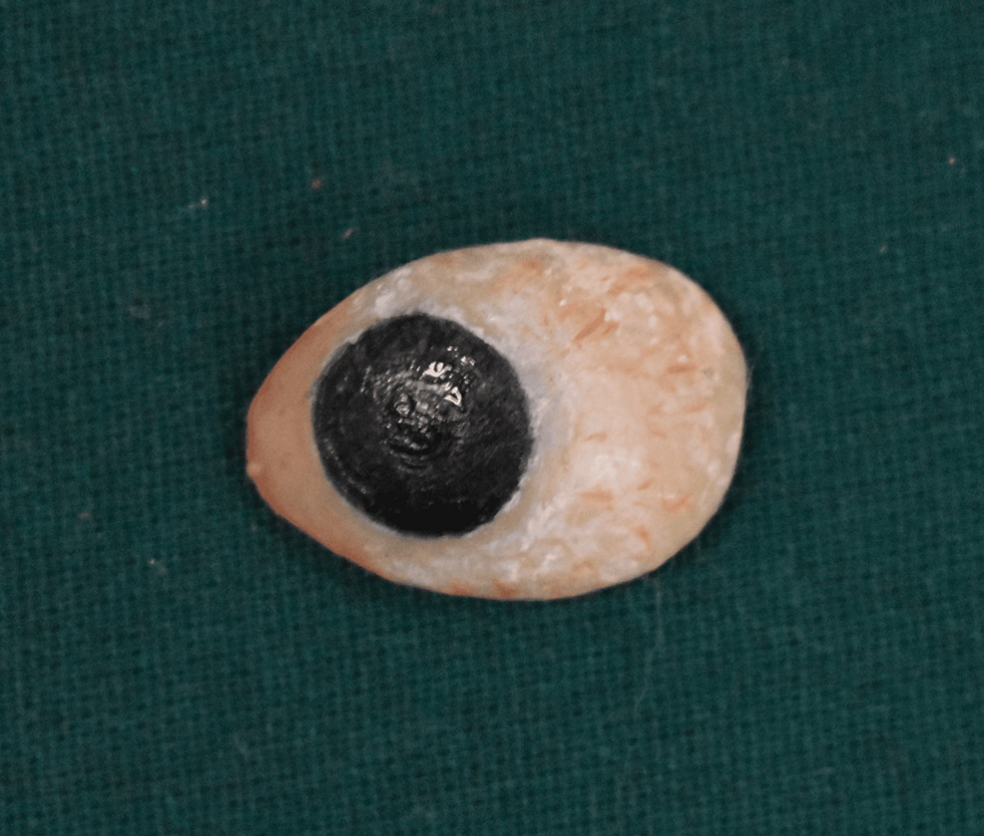
Characterization was done, and a protective G-coat was applied on the prosthesis.

**Figure 11:**
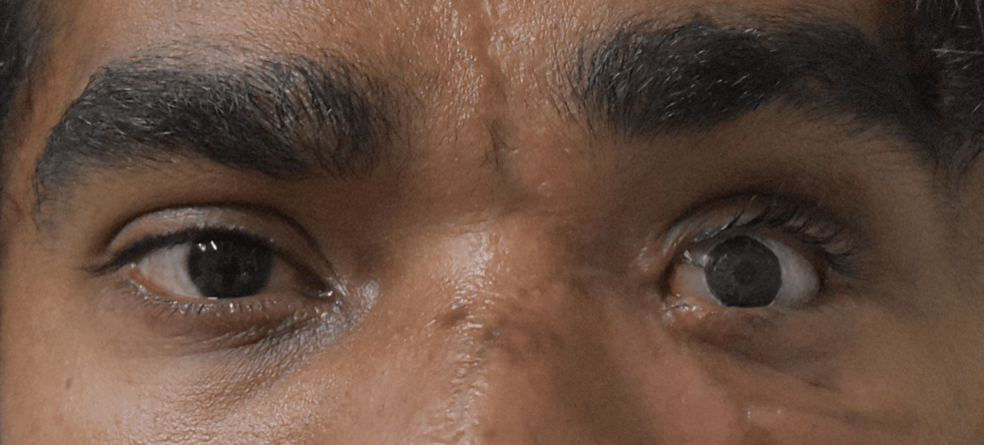
Posttreatment photograph with ocular prosthesis.

Using type III dental stone (Kalabhai, India), the completed wax pattern was invested in a flask. Dewaxing was carried out traditionally. The flask was reopened after and filled with a mixture of clear heat-cure PMMA and teeth molding powder (DPI, India) to fabricate a scleral blank. The manufacturer's instructions were followed while mixing the powder and liquid and were cured following the manufacturer's instructions. The sclera was tinted using composite stains (SR Adoro Stains, Ivoclar Vivadent, Germany), which were then cured in the polymerization unit (Figure 10). A layer of protection (G-Coat Plus, GC America Inc., Alsip, IL, USA) was applied to maintain the characterization. The patient received the finished prosthesis along with postdelivery instructions which included the use of ocular prosthesis for 24 hours to prevent folding of the eyelids and regular cleaning of the ocular prosthesis and eyelids with mild soap and follow-up once a year. The patient was satisfied with the ocular prosthesis (Figure 11).

## Discussion

The eye is the most important facial feature and plays a major role in facial expressions and beauty. Its complex movements and dynamic structure are essential for nonverbal communication and emotional expression. The prosthodontist and surgeon must work together to handle an anophthalmic socket. A complete understanding of anatomy is required for the effective treatment of missing eye rehabilitation because it enables assessment of surrounding structures, which helps to provide proper fit of the ocular prosthesis. Any prosthetic therapy should aim to restore the patient so that they appear normal to the general public in a normal manner. Glass was the preferred material for making ocular prostheses up until the 1940s [6]. The development of PMMA polymers led to their adoption as the preferred material; custom-produced PMMA ocular prostheses provide several benefits, including being nonbrittle, more adaptable, pleasant, aesthetically pleasing, long-lasting, and simple to polish or repair [7].

Similar to the importance of the interpupillary line and horizontal reference planes in tooth restoration, bilateral symmetry is critical in iris alignment. The patient is positioned in their natural head posture (NHP) to capture the horizontal plane for an extraoral maxillofacial prosthesis. NHP is sometimes known as the aesthetic reference point [8]. NHP is the most comfortable head position for a patient to look out to the horizon [9].

Many researchers have suggested several methods to align the iris in the NHP reference plane symmetrically with the adjacent eye. An ocular pupillometer was utilized by Roberts et al. as early as 1969 to align the eye prosthesis [10]. A translucent, bendable graph grid was employed by Guttal et al. and Sinha et al. [11-12]; yet, the grid may result in an incorrect transfer of the iris location. The iris location was previously established using the mediolateral canthi of the eye, and its position was transferred to the ocular stone model using an eyeglass grid. A few authors, including Chamaria et al., Shetty et al., and Arora et al., suggested using a facebow in addition to the scale and graph frame for the iris location [13-15]. Auricular or significant craniofacial abnormalities cannot be treated with this procedure.

The sclera and iris were created by Kale et al. using digital photography, and a clear co-polyester sheet was vacuum-pressed onto the photo paper [16]. Although this technique is difficult and lengthy to learn, the approach successfully replicated the patient's natural eye.

The procedure outlined in this particular case was comparatively easy to use, affordable, and efficient. The challenges faced in this methodology included the difficulty in the placement of the graph sheet on the wax pattern, since the wax pattern had a curved surface. The clinician had a predictable result since the patient's contralateral natural eye was precisely replicated using an iris dimension and the sclera was painted using composite stains.

## Conclusions

Fabricating a custom ocular prosthesis is a difficult process that needs accuracy and precision. Digital photography can improve the effectiveness of this procedure. This article outlines an innovative technique for iris positioning in ocular prostheses utilizing digital photos that can save the clinician's time and energy. This method preserved the wearer's natural look.
